# Plasma Cell-Free DNA and Caspase-3 Levels in Patients with Chronic Kidney Disease

**DOI:** 10.3390/jcm12175616

**Published:** 2023-08-28

**Authors:** Anna Clementi, Grazia Maria Virzì, Sabrina Milan Manani, Massimo de Cal, Giovanni Giorgio Battaglia, Claudio Ronco, Monica Zanella

**Affiliations:** 1 Department of Nephrology and Dialysis, Santa Marta and Santa Venera Hospital, 95024 Acireale, Italy; a.clementi81@virgilio.it (A.C.); giovanni.giorgio.battaglia@hotmail.it (G.G.B.); 2 IRRIV-International Renal Research Institute, 36100 Vicenza, Italy; sabrina.milan@aulss8.veneto.it (S.M.M.); mamo00@libero.it (M.d.C.); cronco@goldnet.it (C.R.); monica.zanella@aulss8.veneto.it (M.Z.); 3 Department of Nephrology, Dialysis and Transplant, St. Bortolo Hospital, 36100 Vicenza, Italy

**Keywords:** cell free DNA, caspase-3, chronic kidney disease

## Abstract

Background: Cell-free plasma DNA (cfDNA) is circulating extracellular DNA arising from cell death mechanisms (apoptosis, necrosis, etc.). It is commonly existent in healthy individuals, but its ranks increase in diverse clinical circumstances, such as kidney disease, sepsis, myocardial infarction, trauma and cancer. In patients with advanced chronic kidney disease, cfDNA is connected to inflammation, and it has been associated with higher mortality. Caspase-3 plays a dominant role in apoptosis, a mechanism of programmed cell death involved in the pathogenesis and progression of chronic kidney disease (CKD). The aim of this pilot study was the evaluation of cfDNA levels and caspase-3 concentrations in patients with chronic kidney disease, in order to investigate the potential role of these molecules, deriving from inflammatory and apoptotic mechanisms, in the progression of renal damage. Methods: We compared cfDNA and caspase-3 levels in 25 CKD patients and in 10 healthy subjects, evaluating their levels based on CKD stage. We also explored correlations between cfDNA and caspase-3 levels in CKD patients and between cfDNA and caspase-3 levels and serum creatinine and urea in this population. Results: We observed that cfDNA and caspase-3 levels were higher in patients with CKD compared to healthy subjects, in particular in patients with advanced renal disease (CKD stage 5). A positive correlation between cfDNA and caspase-3 levels and between cfDNA and caspase-3 and creatinine and urea were also noticed. Conclusions: Patients with chronic kidney disease show higher levels of cfDNA and caspase-3 levels compared to the control group. Based on these preliminary results, we speculated that the worsening of renal damage and the increase in uremic toxin concentration could be associated with higher levels of cfDNA and caspase-3 levels, thus reflecting the potential role of inflammation and apoptosis in the progression of CKD. Future studies should focus on the validation of these promising preliminary results.

## 1. Introduction

Cell-free plasma DNA (cfDNA) is composed of circulating extracellular DNA originating from apoptosis or cell necrosis. This genomic DNA may exist in a cell-free form (mRNAs and miRNAs) or in a mitochondrial DNA (mtDNA). It is generally released from different cellular lines (neutrophils, eosinophils and macrophages), circulating as mononucleosomes, which protect it from degradation. Healthy individuals have small amounts of cfDNA, whose levels increase in the setting of different clinical conditions, such as hemodialysis and peritoneal dialysis [1], sepsis [2,3], myocardial infarction [4], trauma [5] and cancer [6].

Inflammation is a common feature of chronic kidney disease (CKD), especially in end-stage renal disease, and it is related to endothelial dysfunction, oxidative stress and atherosclerotic modifications. In the setting of chronic kidney disease, inflammation appears to play a pivotal role in the release of cfDNA [7]. Moreover, cfDNA has been demonstrated to induce in vitro interleukin-6 (IL-6) production by monocytes [8], thus suggesting a two-way relationship between inflammation and cfDNA in patients with CKD. Furthermore, aerobic exercise has been demonstrated to increase mtDNA independently of inflammatory markers in patients with chronic kidney disease stage 3 and 4 [9]. Additionally, cfDNA levels have been found to be higher in patients undergoing hemodialysis compared to patients with CKD and patients treated with peritoneal dialysis [1].

Finally, increased levels of cfDNA have been demonstrated to be associated with higher mortality in patients with CKD, especially in those undergoing hemodialysis [10].

Apoptosis a homeostatic mechanism responsible for the maintenance of cellular populations in tissues. It may also occur in the setting of immune reactions or noxious agents [11]. Caspases (cysteine aspartases, cysteine–aspartic proteases or cysteine-dependent aspartate-directed proteases) are protease enzymes which play a pivotal role in apoptotic pathway. Their name derives from their specific cysteine protease activity [12]. Most cells have caspases in an inactive proenzyme form, which is activated and induces other pro-caspases, thus initiating a protease cascade. This process is able to amplify the apoptotic signaling pathway and to induce rapid cell death [12]. Three main apoptotic pathways have been described: the extrinsic or death receptor pathway, the intrinsic or mitochondrial pathway, and a pathway involving T-cell mediated cytotoxicity and perforin–granzyme-dependent killing of the cell [12]. These pathways share the execution phase, initiated by the cleavage of caspase-3, resulting in the fragmentation of DNA, degradation of cytoskeletal and nuclear proteins, formation of apoptotic bodies, and expression of ligands finally taken up by phagocytic cells [12]. Therefore, caspase-3 is an effector caspase, responsible for the progression of apoptotic mechanisms.

The aim of this pilot study was the evaluation of cfDNA levels and caspase-3 concentrations in patients with chronic kidney disease, in order to investigate the potential role of these molecules, deriving from inflammatory and apoptotic mechanisms, in the progression of renal damage.

We studied cfDNA levels in patients with CKD and compared their levels with cfDNA in healthy subjects. Furthermore, we evaluated the possible relationship between cfDNA concentrations and the severity of renal disease. Moreover, we analyzed caspase-3 levels in CKD patients and healthy subjects, focusing on its possible change according to CKD stage. Finally, we evaluated the relationship between cfDNA and caspase-3 levels and the correlation between them and uremic toxins (creatinine and urea) in this population of patients.

## 2. Methods

### 2.1. Enrollment and Blood Collection

In this pilot project, we assessed cfDNA levels in 25 CKD patients. In particular, we collected blood samples from 25 patients with chronic kidney disease (5 for each stage: G1-G2-G3-G4-G5) coming to our center (Nephrology Department at San Bortolo Hospital in Vicenza, Italy) for a routine check-up. Patients with infections, autoimmune disease, cancer, or pulmonary or hepatic disease were excluded from the study. CKD G5 on dialysis were excluded as well. Serum creatinine (SCr) was evaluated using an enzymatic method, isotope dilution mass spectrometry traceable by an automatic analyzer (Dimension Vista; Siemens Healthcare, Tarrytown, NY, USA), and eGFR was calculated by the application of the CKD-EPI equation. Biochemical parameters, such as blood urea, sodium and potassium were evaluated by standard laboratory techniques using an automatic analyzer (Dimension Vista, Siemens Healthcare, Tarrytown, NY, USA). Hemoglobin and platelet count (PLT) were measured using the automated hematology analyzer XN 9000 (SYSMEX, Kobe, Japan). Comorbidities, such as diabetes, hypertension and cardiovascular disease (CVD), were recorded for all patients.

Patients’ clinical characteristics and laboratory parameters were registered for all subjects. In addition, 10 healthy volunteers (CTR) were included as control group. Blood from these subjects was kindly provided by the blood bank of St. Bortolo Hospital. Healthy volunteers with any condition influencing kidney function were excluded from the study to avoid confusion of the data.

### 2.2. Biological Sample Collection

Blood samples were collected from CKD patients and healthy controls into EDTA tubes. All samples were centrifuged for 10 min at 3500 rpm. Plasma was carefully removed without touching the cell pellet and re-centrifuged at 13,000 rpm for 15 min. After centrifugation, the supernatant was placed into a clean tube.

### 2.3. Isolation and cfDNA Extraction

Within the first 30 min after drawing fresh plasma samples from all patients, we performed DNA isolation and extraction. DNA was isolated from 250 µL of plasma by a specific DNA isolation kit (ArrowDNA Kit; NorDiag, Holliston, MA, USA) through automatic extractor NorDiag Arrow (NorDiag, Holliston, MA, USA), as per the manufacturer’s protocol. The DNA was eluated in 35 µL of elution buffer. A Picodrop Spectrophotometer (Bulldog Bio, Portsmouth, NH, USA)was used to evaluate cfDNA concentration after extraction.

### 2.4. Real-Time Quantitative PCR

Plasma cfDNA was measured via real-time quantitative assay using RotorGene 6000 (real-time cycler with rotary analysis) (Qiagen, Milan, Italy) for the β-actin gene. We reported the primers sequence used for β-actin gene as: Forward (5′–3′) GCGCCGTTCCGAAAGTT; Reverse (5′–3′): CGGCGGATCGGCAAA.

A volume of 10 µL of cfDNA was added to each reaction mixture (final volume of 15 μL). The real-time reaction mix consisted of 4.95 μL of deionized water, 1.25 μL of Eva Green (intercalating double strand (ds) DNA-binding dye) (Biotium, Hayward, CA, USA), 5 μL of Real Time PCR Buffer, magnesium free (TakaRa, Shiga, Japan), 1.5 μL of Magnesium (TakaRa, Shiga, Japan), 0.5 μL of dNTP Mixture (TakaRa, Shiga, Japan), 5 μM of each primer, and 0.3 μL of Takara Ex Taq R-PCR (TakaRa, Shiga, Japan).

The real-time protocol of each amplification and each assay was composed by different steps, including an initial activation step at 95 °C for 3 min followed by 50 amplification cycles of 30 s denaturation, 30 s annealing at 65 °C (touchdown 1 °C for 10 cycles) and 30 s elongation, and a final elongation step at 72 °C for 5 min. The fluorescence acquisition was performed on the green channel. The real-time PCR products were heated to 99 °C and cooled for heteroduplex formation (the double-stranded molecule of DNA originated through the genetic recombination of single complementary strands), and melt was monitored by fluorescence emission through appropriate denaturation range (50–99 °C). Melt-curve analysis, evaluating the dissociation characteristics of double-stranded DNA during heating, showed a single product-specific melting temperature with a mean of 93.5 °C for β-actin gene.

For each run, one aliquot of stock DNA was serially diluted with deionized water to generate a 6-point standard curve (200000-20000-2000-200-20-2 pg/mL). A conversion factor of 6.6 pg of DNA per diploid cell was used for calculation. Results are expressed as genome equivalents (GE)/mL; 1 GE/mL equals 6.6 pg DNA.

We performed real time runs in two different times (different real-time mixture and different work section) in triplicate with samples and standard curves. A blank reaction and multiple negative controls were included in every run.

### 2.5. Determination of Caspase-3 Activity

We performed an enzyme-linked immunosorbent assay (ELISA) test to evaluate caspase-3 levels in the plasma. The ELISA assay is an immunological assay commonly used to measure antibodies, antigens, proteins and glycoproteins in biological samples. This test is based on a solid-phase type of enzyme immunoassay to detect and measure the presence of a specific ligandin a liquid biological material using antibodies directed against the protein to be measured. Caspase-3 concentration was measured by Human Caspase-3 Instant Enzyme-Linked Immuno-Sorbent Assay (ELISA) Kit (eBioscience, San Diego, CA, USA) via a fluorometric assay with detection setting at 450 nm by VICTORX4 Multilabel Plate Reader (PerkinElmer Life Sciences, Waltham, MA, USA). Each experiment was performed in triplicate. Caspase-3 concentrations (ng/mL) were calculated from the standard curve (10-5-2.5-1.25-0.63-0.31-0.16 ng/mL) as per the manufacturer’s protocol. Human Caspase-3 Instant ELISA Kit sensitivity reported for this ELISA test is 0.12 ng/mL.

### 2.6. Statistical Analysis

Statistical analysis was performed with the SPSS Software package and Excel. A *p*-value of <0.05 was considered statistically significant. Results are presented as percentages, or media and standard deviation (parametric variables) or medians and interquartile ranges (nonparametric variables). In order to compare two groups, we used the Mann–Whitney U test or *t*-test, as appropriate. In case of three or more groups, we used the Kruskal–Wallis test or ANOVA test for multiple comparisons, as appropriate. Correlation coefficients were evaluated by different statistic tests (the Spearman’s rank or Pearson’s test), as appropriate.

We applied a linear transformation of cfDNA to manage the data better.

## 3. Results

In our study, a total of 25 patients with chronic kidney disease were included. A total of 14/25 patients were male. The mean age of enrolled patients was 57 ± 17 years old.

The causes of end-stage renal disease (ESRD) were diabetic nephropathy (seven patients), hypertension (eight patients), nephroangiosclerosis (three patients), polycystic kidney disease (two patients), other causes (three patients), or unknown causes (two patients).

For our proposes, we divided patients according to CKD stage (G1-G2-G3-G4-G5). Each stage was composed of five patients. No G5 patients were on dialysis. Table 1 reports comorbidities and biochemical parameters for each stage of CKD (Table 1). Biochemical parameters of healthy subjects were within the range. No comorbidities were found.

**Figure 1:**
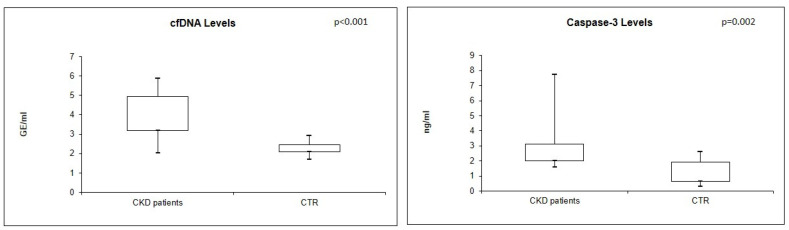
cfDNA and caspase-3 levels in CKD patients compared with healthy individuals (Mann–Whitney U test).

Real-time quantitative analysis showed significantly higher levels of plasma cfDNA and caspase-3 in CKD patients compared with CTR (both *p* < 0.05) (Figure 1).

In particular, the median value of cfDNA resulted 37,537 GE/mL (IQR: 1495.4–90,326.09) in CKD patients, compared to 186.7 GE/mL (IQR 122.95–285.8) in controls. In the same way, the median value of caspase-3 resulted 2.20 ng/mL (IQR: 1.99–3.12) in CKD patients, compared to 1.114 ng/mL (IQR: 0.6–1.9) in healthy individuals.

Furthermore, we analyzed cfDNA and caspase-3 levels in CKD patients with different comorbidities. Median values of cfDNA and caspase-3 did not differ significantly between patients with (n = 4) and without diabetes (cfDNA *p* = 0.20, caspase-3 *p* = 0.37), with (n = 13) hypertension and without (cfDNA *p* = 0.11, caspase-3 *p* = 0.21), with (n = 5), with cardiovascular comorbidity and without (cfDNA *p* = 0.17, caspase-3 *p* = 0.35).

**Figure 2:**
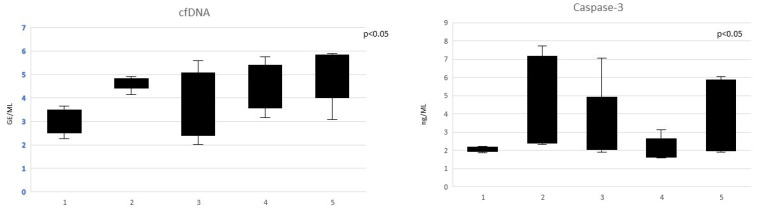
cfDNA and caspase-3 levels in CKD patients according to the stage of the disease (Kruskal–Wallis test).

Furthermore, we analyzed cfDNA and caspase-3 levels in each stage of CKD. cfDNA and caspase-3 levels were significantly different between CKD stages (both *p* < 0.05) (Figure 2). In particular, cfDNA and Capsase-3 values were lower in patients with CKD stage G1, while they resulted higher in patients with CKD stage G5. Furthermore, cfDNA and caspase-3 levels did not differ significantly between CKD G1 and CKD G2 and CKD G3. cfDNA and caspase-3 values resulted significantly higher in CKD G5 compared with all CKD stages (*p* = 0.04 and *p* = 0.05, respectively).

**Figure 3:**
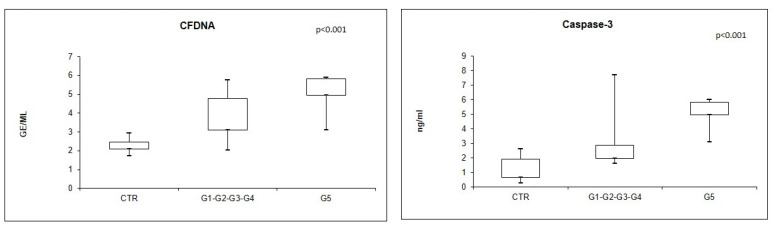
cfDNA and caspase-3 levels in CKD G5 patients compared with CKD G1-G2-G3-G4 patients and healthy individuals (Kruskal–Wallis test).

In addition, we dived CKD patients in two groups (G1-G2-G3-G4 vs. G5) to better understand the contribution of CKD on cfDNA and caspase-3 levels. In particular, CKD patients in G5 have significantly higher levels of cfDNA and caspase-3 levels compared to controls and G1-G2-G3-G4 patients (*p* < 0.001) (Figure 3).

In addition, we investigated the relationship between cfDNA, caspase-3, urea and serum creatinine. A very strong significant positive correlation was observed between cfDNA and caspase-3 levels (Sperman’s rho = 0.7, *p* < 0.001). In addition, positive correlations were observed between urea/cfDNA (Sperman’s rho = 0.57, *p* = 0.01) and urea/caspase-3 (Sperman’s rho = 0.53, *p* = 0.02), serum creatinine/cfDNA (Sperman’s rho = 0.49, *p* = 0.03) and serum creatinine/caspase-3 (Sperman’s rho = 0.40, *p* = 0.05). We summarized correlation data in Table 2.

## 4. Discussion

In our pilot study, we evaluated cfDNA levels and caspase-3 concentrations in patients with chronic kidney disease, in order to investigate the potential role of these molecules, deriving from inflammatory and apoptotic mechanisms, in the progression of renal damage.

cfDNA has been isolated from the blood and urine of healthy subjects, but its levels increase in the setting of sepsis [2,3], myocardial infarction [4], trauma [5] and cancer [6]. Also, patients with chronic kidney disease undergoing peritoneal dialysis and hemodialysis have been reported to have increased levels of cfDNA [1].

In particular, compared with concentrations before hemodialysis session, higher cfDNA levels have been found during the treatment, decreasing to the pre-dialysis concentrations within 30 min after the procedure [13].

Moreover, in a recent study performed on patients undergoing hemodialysis, higher cfDNA levels have been demonstrated to predict poor outcome in this population of patients [14]. Specifically, the authors have found a positive correlation between cfDNA levels and white cells’ count, IL-6 and C-reactive protein. Deceased patients with end-stage renal disease presented higher levels of both cfDNA and inflammatory markers (IL-6 and C-reactive protein), compared with living subjects [15].

Increased concentrations of mitochondrial DNA have also been detected in patients undergoing hemodialysis, together with altered mitochondrial homeostasis [14]. Unlike genomic DNA, mitochondrial DNA is similar to bacterial DNA, so it may behave as a damage-associated molecular pattern (DAMP). Therefore, it has been recently proposed as a crucial inflammatory marker playing an important role in the pathogenesis of microvascular inflammation in hemodialysis patients [14]. The authors have demonstrated a relationship between mtDNA and cardiovascular events in this population of patients [14].

According to these data, in our study, cfDNA levels were higher in CKD patients compared to healthy subjects. Moreover, its levels increased in patients with CKD stage G5 compared to all CKD stages. These preliminary data suggest a possible relationship between cfDNA release and the progression of renal disease. Indeed, in a recent study, cfDNA has been demonstrated to be closely associated with diabetic kidney disease, predicting its worsening in patients with type 2 diabetes [16]. Indeed, higher levels of cfDNA were related to the annual decline in estimate glomerular filtration rate (eGFR), independently of age, blood pressure, glucose and duration of diabetes. Moreover, patients with diabetic kidney disease had higher serum levels of cfDNA than patients without kidney damage. Diabetic retinopathy was also associated with increased concentrations of cfDNA [16].

Furthermore, a previous study analyzed the role of cfDNA in the setting of acute kidney injuries induced by ischemia, suggesting its possible effect in the amplification of renal damage [17]. Recently, extracellular DNA has also been found to represent an early and sensitive marker of acute kidney injury in an animal model of glycerol-induced nephropathy [18]. In this study, the authors demonstrated an earlier rise in extracellular DNA levels compared to creatinine and urea concentrations following glycerol nephropathy. Indeed, uremic toxins significantly increased 24 h after the glycerol injection, while extracellular DNA levels rose 1 to 3 h after the model induction [18].

The role of cfDNA has also been investigated in kidney transplantation. In case of graft cell death (induced by rejection, acute tubular necrosis, infection and ischemia), cfDNA concentrations early increase. The monitoring of its levels may help clinicians to prevent premature graft loss, in addition to personalize immunosuppression therapy [19].

In our study, hypertension, diabetes and cardiovascular comorbidities did not affect cfDNA concentrations, which may increase as the result of the contemporaneous presence of all these factors. Our preliminary data have also demonstrated a positive correlation between cfDNA and creatinine and urea, thus suggesting an increased cfDNA release secondary to the uremic status. Urea, as well as other uremic toxins (indoles, p-cresyl sulfate and phosphate), is known to induce oxidative stress by promoting the production of reactive oxygen species (ROS) and leukocyte free radicals, thus resulting in endothelial dysfunction and apoptosis [20]. Both mechanisms may be involved in the increased release of cfDNA.

We have also found higher levels of caspase-3 in patients with chronic kidney disease compared to healthy subjects, and its concentrations increased with the worsening of renal damage. Indeed, caspase-3 levels were higher in patients with CKD stage G5, like cfDNA concentrations. Moreover, a positive correlation was found between caspase-3 levels and creatinine and urea concentrations. A relationship between cfDNA and caspase-3 levels was observed as well.

In higher organisms, apoptosis is known to be one of the main sources of cfDNA, and to be mediated by caspases, a group of cysteine proteases cleaving after an aspartate residue in their substrates [21]. In particular, caspase-3 plays a pivotal role in the in the execution pathway of apoptosis, which is one of the main mechanisms involved in the progression of renal disease, together with endothelial dysfunction [12,20]. Furthermore, it is known that elevated urea levels can lead to molecular changes responsible for the generation of reactive oxygen species and apoptosis. Also, hemodialysis is known to induce apoptosis of leukocytes, as shown by the typical ladders which appear after electrophoresis [22].

Therefore, it is clear that cfDNA and caspase-3 levels are tightly connected in the setting of renal disease progression, and their concentrations increase with the severity of kidney damage, thus reflecting the effect of the uremic toxins. Indeed, our data suggest that inflammation and apoptosis may be responsible for the higher levels of cfDNA in patients with chronic kidney disease.

Our study has some limitations. First, this is a pilot study whose population consists of 25 CKD patients, five patients for each stage of CKD; second, we evaluated caspase-3 concentrations, whose role is fundamental in the execution pathway, therefore we are not able to indicate the specific apoptotic mechanism activated in this population of patients; third, we have not investigated the possible role of autophagy in the progression of CKD. Future studies are needed to validate and confirm these promising preliminary results and to investigate the role of other caspases in the progression of chronic kidney disease.

## 5. Conclusions

cfDNA and caspase-3 concentrations are higher in patients with chronic kidney disease compared with healthy subjects, and their levels increase with the worsening of renal damage. They are tightly related, and they reflect the activation of apoptotic and inflammatory mechanisms playing a pivotal role in the pathogenesis of CKD. Future studies should focus on the validation of these promising preliminary results, and they should analyze the specific apoptotic and/or autophagy pathways that may be involved in chronic kidney disease.

## Figures and Tables

**Table 1:** Comorbidities and biochemical parameters in study population.

	Age, Years	Diabetes	Hypertension	CVD	Serum Creatinine (µmol/L)	Urea (mmol/L)	Sodium (mmol/L)	Potassium (mmol/L)	Hb (g/dL)	PLT (×10.9/L)
G1	42, 19–43	0	1	0	84.95, 84.05–89.35	5.21, 5.03–5.48	139.5, 139–141	4.1, 4.0–4.3	14.6, 14,38–14.92	223.5, 209.5–248.8
G2	55, 44–72	1	3	1	93.7, 83.8–103.6	8.3, 7.6–9.05	138.5, 138–138.8	3.8, 3.7–3.9	14.95, 14.72–15.18	319, 260–377
G3	69, 59–76	1	3	2	134.6, 112.6–169.8	8.47, 8.22–9.96	142, 141–142	4.0, 4.0–4.1	14.3, 13.8–14.9	246, 205–287
G4	50, 46–74	0	3	2	223.5, 205.1–248.6	14.46, 13.51–15.45	141.5, 141–142	3.9, 3.7–4.1	11.8, 11.5–12.1	217.5, 204–245.5
G5	69, 69–71	2	3	0	444.4, 440.1–466.4	25.4, 23.4–25.4	139, 139–142	4.0, 3.9–4.5	12.3, 11.7–12.4	202, 190–208

**Table 2:** Summary of correlations.

	Spearman’s rho Correlation	*p*
cfDNA/Caspase-3	0.7	<0.001
Urea/cfDNA	0.57	0.01
Urea/Caspase-3	0.53	0.02
Serum creatinine/cfDNA	0.49	0.03
Serum creatinine/Caspase-3	0.40	0.05

## Data Availability

All data generated or analyzed during this study are included in this article. Further inquiries can be directed to the corresponding author
